# 383. VA SHIELD: Building an Interactive Dashboard for Surveillance of Infectious Diseases across the Veterans Health Administration, 2000 – 2023

**DOI:** 10.1093/ofid/ofae631.118

**Published:** 2025-01-29

**Authors:** Lauren Epstein, Ariana Paredes-Vincent, Christopher Ogston, Maria C Rodriguez-Barradas, Robert A Bonomo, Christopher Woods, Sheldon T Brown

**Affiliations:** Atlanta VA, Atlanta, Georgia; Veterans Health Administration, New York, New York; Veterans Health Administration, New York, New York; Michael E. DeBakey VAMC and Baylor College of Medicine, Houston, Texas; Case Western Reserve University/ Louis Stokes Cleveland VA Medical Center, Cleveland, OH; Durham VA Medical Center/DUke, Durham, North Carolina; James J Peters VAMC, Bronx, NY

## Abstract

**Background:**

The Veterans Health Administration (VA) utilizes a comprehensive data system that can be updated in real time to help detect emerging diseases across the United States. We describe a novel interactive dashboard that was developed to track emerging infections across the VA system.

**Figure 1.**
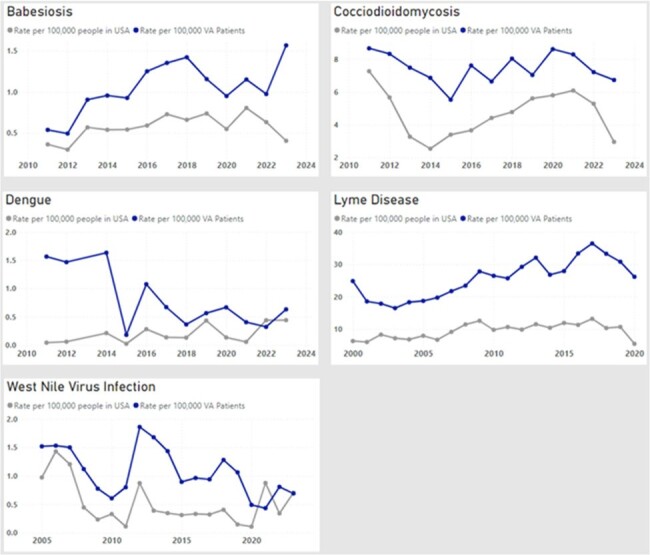
Comparison of trends of selected Infectious Diseases, VA system vs. U.S. population (2000 – 2023).

**Methods:**

This project was developed as part of VA Science and Health Initiative to Combat Infectious and Life-Threatening Diseases (VA SHIELD), a comprehensive biorepository of specimens and clinical data that was developed in 2020 to address national health threats across the VA system to improve diagnostic and therapeutic capabilities.

We compiled a list of rare and emerging infectious diseases and identified appropriate ICD-9 and ICD-10 codes for each infectious disease based on expert opinion. All unique persons that received care at any type of VA facility (inpatient, outpatient) were eligible to be included in the analysis. We calculated incidence rates (per 100,000 VA population) based on the number of unique patients that received care at VA facilities annually from 2000 – 2023. We compared VA rates of selected infectious diseases with national rates using publicly available data from CDC’s WONDER database, where available.

**Figure 2.**
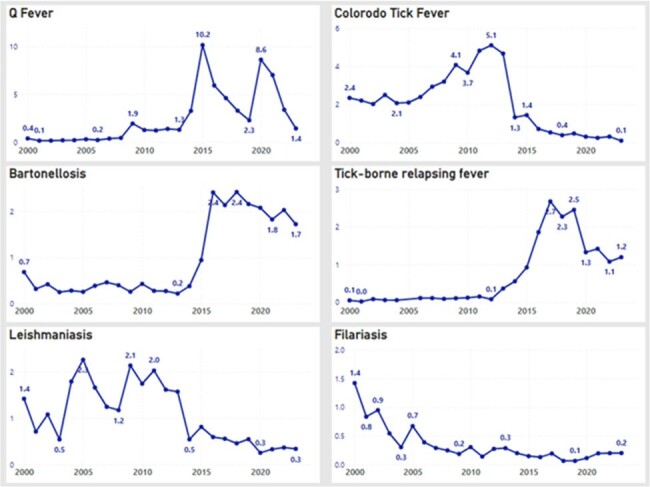
Incidence of rare Infectious Diseases detected across the VA system (no CDC comparison), 2000 – 2023. Rate per 100,000 VA patients.

**Results:**

We included 47 unique infectious diseases in our assessment; 16 (34%) were also included in CDC’s WONDER database. Compared to CDC’s WONDER database, incidence rates and geographic trends of Coccidioides, Babesiosis, Dengue, Lyme disease and West Nile in VA population were similar to trends across the U.S. population (Figure 1). Among diseases that are not reported in CDC’s WONDER database, the incidence of Leishmaniasis increased in 2005 across the VA population while Bartonellosis, Q fever, and Tick-borne relapsing fever incidence increased in 2015 (Figure 2).

**Conclusion:**

We illustrate how a VA dashboard demonstrates infectious disease trends across the U.S. Although some infections may be Veteran specific and related to deployment by military Reservists, others reflect overall population trends. Linkage of the dashboard to a structured surveillance program can serve as a powerful investigational tool to evaluate emerging outbreaks and trends due to environmental exposures across the U.S.

**Disclosures:**

**All Authors**: No reported disclosures

